# A single-tube allele specific-polymerase chain reaction to detect T315I resistant mutation in chronic myeloid leukemia patients

**DOI:** 10.1186/1756-8722-4-7

**Published:** 2011-02-08

**Authors:** Wanwisa Wongboonma, Wanna Thongnoppakhun, Chirayu U Auewarakul

**Affiliations:** 1 Department of Immunology, Faculty of Medicine Siriraj Hospital, Mahidol University, Bangkok 10700, Thailand; 2 Department of Research and Development, Faculty of Medicine Siriraj Hospital, Mahidol University, Bangkok 10700, Thailand; 3 Department of Medicine, Faculty of Medicine Siriraj Hospital, Mahidol University, Bangkok 10700, Thailand

## Abstract

**Background:**

*BCR-ABL *kinase domain (KD) mutation is the major mechanism contributing to suboptimal response to tyrosine kinase inhibitors (TKI) in *BCR-ABL*-positive chronic myeloid leukemia (CML) patients. T315I mutation, as one of the most frequent KD mutations, has been shown to be strongly associated with TKI resistance and subsequent therapeutic failure. A simple and sensitive method is thus required to detect T315I mutation at the earliest stage.

**Methods:**

A single-tube allele specific-polymerase chain reaction (AS-PCR) method was developed to detect T315I mutation in a mixture of normal and mutant alleles of varying dilutions. Denaturing high performance liquid chromatography (DHPLC) and direct sequencing were performed as a comparison to AS-PCR.

**Results:**

T315I mutant bands were observed in the mixtures containing as low as 0.5-1% of mutant alleles by AS-PCR. The detection sensitivity of DHPLC was around 1.5-3% dilution whereas sequencing analysis was unable to detect below 6.25% dilution.

**Conclusion:**

A single-tube AS-PCR is a rapid and sensitive screening method for T315I mutation. Detection of the most resistant leukemic clone in CML patients undergoing TKI therapy should be feasible with this simple and inexpensive method.

## 1. Background

Chronic myeloid leukemia (CML) is a chronic hematopoietic stem cell disorder characterized by extensive proliferation and expansion of myeloid cells at varying stages of maturation and differentiation [1]. The hallmark of CML is the Philadelphia (Ph) chromosome which occurs as a result of a reciprocal chromosomal translocation between chromosomes 9 and 22, creating a new fusion gene, *BCR-ABL*, with constitutive tyrosine kinase activity [2]. Targeting *BCR-ABL*- transfected cell lines and murine CML models with a variety of tyrosine kinase inhibitors (TKI) has led to a landmark discovery of a novel *BCR-ABL *targeting drug, imatinib, which subsequently entered clinical trials, showed significant clinical benefits and has become a standard of care for CML patients worldwide [1,3-5].

Unfortunately, failure to respond to imatinib developed in some CML patients as a result of resistant mutations arising in the *BCR-ABL *kinase domain (KD), leading to shortened survivals of CML patients with these mutations as contrasted to those without [6-11]. The frequency of KD mutations varied from 30% to 50% depending on the studied CML cohorts and the sensitivity and specificity of the detection methods [10-16]. The majority of mutations in imatinib-resistant patients usually occurred within the nine amino acid positions of KD including G250E, Y253H/F, E255K/V, T315I, M351T, F359V, and H396 with varying sensitivities to TKI [17-21]. One of the most common mutations, T315I, is associated with the most resistance to TKI, not only to the 1^st ^generation TKI such as imatinib, but also to the newly approved 2^nd ^generation TKI such as nilotinib and dasatinib [9,10,17,21-23]. Screening for T315I mutations is now recommended for all CML patients undergoing TKI treatment and should be performed as early as possible to detect the lowest levels of the mutant clone [24,25].

In this study, we set out to develop a single-tube allele specific-polymerase chain reaction (AS-PCR) to identify the most resistant KD mutation, T315I, in Thai CML patients. Denaturing high performance liquid chromatography (DHPLC) and sequencing analysis were also performed as a comparison to AS-PCR. We found that our method is simple, rapid, and inexpensive and thus suitable for routine use, especially for CML patients residing in the developing worlds.

## 2. Methods

### 2.1 Preparation of RNA and cDNA template

Total RNA was extracted from leukocytes using TRIzol^® ^reagent (Invitrogen, CA, USA). Complementary DNA (cDNA) was generated by SuperScript III cDNA synthesis kit (Invitrogen, CA, USA) following the manufacturer's instructions. BA/F3 cell lines expressing the wild-type (WT) full-length *BCR-ABL *fusion gene and T315I mutant cell lines were courteously provided by the Oregon Health & Science University [5]. RNA from T315I mutant cell lines was serially diluted by WT BA/F3 cells to prepare 10 dilutions with indicated percentages of T315I mutants. Thirty RNA samples from non-leukemic patients were also used as negative control samples to optimize the AS-PCR condition.

### 2.2 Detection of T315I mutation by AS-PCR

AS-PCR was performed using three primer pairs consisting of 1) T315I mutant primers, forward primer (MT_F) (5'-GCCCCCGTTCTATATCATAAT-3') and reverse primer (MT_R) (5'-GGATGAAGTTTTTCTTCTCCAG-3'), which was adapted from the previously published primer set [20,26], 2) the WT primers, WT_F (5'-TGGTTCATCATCATTCAACGGTGG-3') and WT_R (5'-GTTCCCGTAGGTCATGAACTCAG-3'), and 3) internal control primers, forward (β-actin_F) (5'-gtggggcgccccaggcacca-3') and β-actin_R (5'-gtccttaatgtcacgcacgatttc-3') [27]. First, the AS-PCR was optimized by varying annealing temperature (Ta) (55° to 62°C), MgCl_2 _concentration (1.0-2.5 mmol/L), and primer ratios (MT: WT ratio of 8:2, 7:3, 6:4, and 5:5). Briefly, the optimized condition was performed in a 25-μL mixture of 1 μL cDNA, 2.5 mmol/L MgCl2, 0.2 mmol/L of each dNTP, 3% DMSO, and 0.625 unit of *Taq *DNA polymerase (Invitrogen, USA) together with 14 pmol of MT primers, 6 pmol of WT primers, and 1 pmol of β-actin primers. The PCR profile was as follows: initial denaturation at 95°C for 5 minutes (min), followed by 35 cycles of denaturation at 94°C for 45 seconds (sec), annealing at 57°C for 30 sec, extension at 72°C for 1 min, and final extension at 72°C for 5 min. PCR products of T315I mutant, T315WT, and β-actin were 158 bp, 374 bp, and 540 bp, respectively. The products were assessed on a 2% agarose gel and staining with ethidium bromide. Thirty RNA sample from non-leukemic patients were used as negative control samples to optimize AS RT-PCR conditions for T315I.

### 2.3 Detection of *BCR-ABL *KD mutation by DHPLC and DNA sequencing

The primary PCR step was performed using a pair of primers designed to cover *BCR-ABL *gene. Two micrograms of cDNA template were amplified in a total volume of 20 μL with the following constituents, 0.2 U of high-fidelity DNA polymerase (Phusion™, FINNZYME), 5X Phusion buffer, 2 mmol/L of MgCl_2_, 0.2 mmol/L of each dNTPs, 10 pmol of each primers (forward primers: B2A _f 5'-acagcattccgctgaccatcaataag-3' and reverse primer: BA_r 5'-atggtccagaggatcgctctct-'3) [8]. The reaction mixture was placed in a thermal cycler (Veriti, Applied Biosystems, CA) under the PCR profile as follows: initial denaturation at 98°C for 30 sec, 35 cycles of amplification (at 98°C for 10 sec, 60°C for 30 sec, and 72°C for 1 min 30 sec), and a final extension at 72°C for 10 min. The product band of 1,643 bp (B2A2) or 1,719 bp (B3A2) was visualized on ethidium bromide-stained 1.5% agarose gel. A secondary PCR step for amplification of KD amino acid codon 206-428 using the internal two primer pairs, were designed to amplify two partially overlapping fragments consisting of fragment 1 forward primers: abl_1F (5'-tggttcatcatcattcaacggtgg-3') and reverse primers: abl_1R (5'-tctgagtggccatgtacagcagc-'3), and fragment 2 forward primers: abl_2F (5'-tcatgacctacgggaacctc-3') and reverse primers: abl_1R (5'-atactccaaatgcccagacg-'3). The PCR reaction was performed in a volume of 50 μL containing 2 μL first round PCR product, 0.2 U of high-fidelity DNA polymerase, 1.5 mmol/L of MgCl_2_, 0.2 mmol/L of each dNTPs, and 15 pmol of each primers. The PCR profile was as follows: initial denaturation at 98°C for 30 sec, 35 cycles of amplification (98°C for 10 sec, 60°C for 30 sec, 72°C for 40 sec), and final extension at 72°C for 5 min. The fragment 1 (447 bp) and fragment 2 (333 bp) PCR products were assessed and prepared for further analysis by DHPLC and sequencing.

Prior to DHPLC analysis, mutant products were mixed with WT in a 1:1 ratio and denatured by heating at 95°C for 5 min followed by gradual cooling at 1°C/min to 25°C within 70 min in order to allow heteroduplex and homoduplex formation [28]. DNA were analyzed using a WAVE^® ^nucleic acid fragment analysis system (Transgenomic Inc, Omaha, NA, USA) by injection of 5 to 10 μL of each fragment onto a chromatography column (DNASep HT column, Transgenomic, USA) and were then eluted at 59°C with a linear acetonitrile gradient in 0.1 M triethylammonium acetate buffer (TEAA) at pH 7.0. The eluted cDNA was detected by 260 nm UV absorbance. For sequencing, the PCR products were purified using the Qiaquick PCR purification kit (Qiagen, USA) or ExoSAP-IT^® ^(GE Healthcare Bio-Sciences, USA), following the manufacturer' s protocol. Sequencing with forward and/or reverse primers in secondary PCR steps was carried out by the ABI3730XL DNA analyzer (Applied Biosystems, USA) using ABI BigDye terminator cycle sequencing kits (Applied Biosystems, USA). The results were compared with the WT *ABL1 *(accession no. NM_005157.3).

Sensitivity of DHPLC and direct sequencing methods were evaluated by determination of dilutions of known quantities of T315I mutant products and WT products with indicated percentages of mutant products.

## 3. Results

### 3.1. Detection of T315I mutation in dilution mixtures by AS-PCR

**Figure 1:**
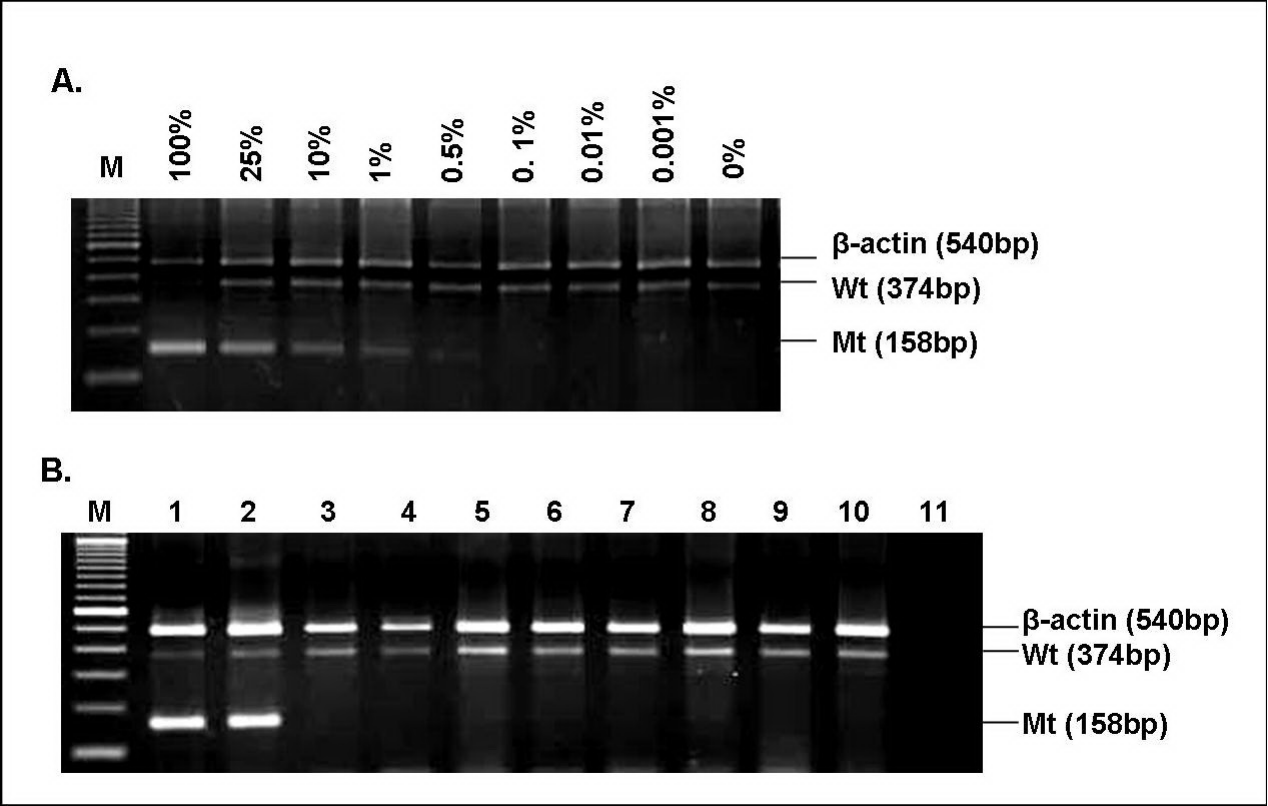
**Sensitivity of T315I mutation detection by AS-PCR method **. Serial dilutions of T315I mutants with wild-type cells demonstrates 158-bp mutant bands in 100%, 25%, 10%, 1%, and 0.5% mixtures (**Figure 1A**); Representative samples of T315I mutated cell lines (Lane 1), T315I mutated CML case, (Lane 2), seven non-mutated non-leukemic cases (Lanes 3-9), and BA/F3 cell lines (Lane 10) are shown in **Figure 1B**; Lane 11, blank; Lane M, a 100-bp DNA marker.

AS-PCR was designed to specifically detect T315I mutations using the cDNA templates synthesized from RNA with known percentages of the mutant allele. Mutants, WT, and internal controls could be detected in a single reaction. We first optimized the annealing temperature and the ratio of each primer pair and the results of our triplicate independent experiments demonstrated that the optimal annealing temperature was 57°C with the primers ratio of 7:3:0.5 of mutants, WT, and internal control primer pairs, respectively. A 158-bp PCR product was derived from the mutant allele whereas a 374-bp PCR product could represent a heterozygous allele or no mutant allele and a 540-bp product was an internal control. In three independent experiments, a strong T315I mutant band was detected in as low as 1% dilution and a faint band was observed in 0.5% dilution (Figure 1). In addition, 30 samples from non-leukemic patients and WT BA/F3 cell lines were also tested to ensure our AS-PCR's specificity; all of which were found negative for T315I, therefore, BA/F3 cell lines were subsequently utilized as a T315I negative control.

### 3.2 Detection of T315I in dilution mixtures by DHPLC and sequencing

**Figure 2:**
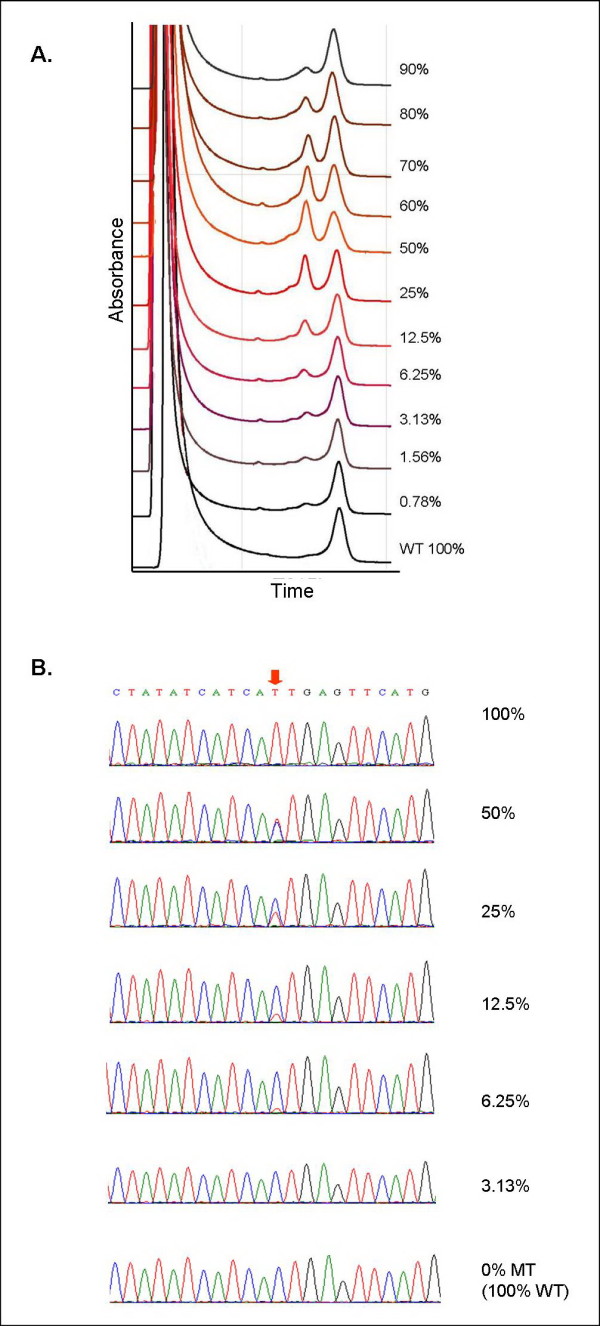
**Sensitivity of T315I mutation detection by DHPLC and sequencing analysis **. DHPLC chromatogram patterns generated by each mutant allele concentration are shown in **Figure 2A **and sequencing results corresponding to each DHPLC-generated chromatogram are shown in **Figure 2B**; Red arrow indicates c.947 C > T mutation; C, Wild-type; T, Mutant.

DHPLC was performed first as a screening to detect abnormal peaks on chromatograms. The heteroduplexes generated from the heterozygous products gave a peak that was distinctive from the WT. Abnormal peaks could be clearly detected in 90%, 80%, 70%, 60%, 50%, 25%, 12.5% 6.25%, 3.13%, and 1.56% dilutions (Figure 2A). An ambiguous peak was also seen at 0.78% dilution. Prior to DHPLC analysis, PCR products were mixed with a WT product in a 1:1 ratio to prevent the false negative results as the homoduplexes derived from the homozygous mutant cDNA (100%MT) generated a sharp peak that was quite similar to the homozygous WT peak (0% MT). T315I peak had a different peak pattern from other common mutations (Y253F, Y253H, E255K, M351T, and F359V) (data no shown).

For direct sequencing, a "T" peak indicated the presence of T315I which could be clearly seen in 100%, 50%, 25%, 12.5%, and 6.25%. A "C" peak which represented the WT *BCR-ABL KD *allele could be seen in 50%, 25%, 12.5%, 6.25%, 3.13%, and 0% dilution (100% WT) (Figure 2B).

### 3.3 Detection for T315I in DHPLC and sequencing positive patients

**Figure 3:**
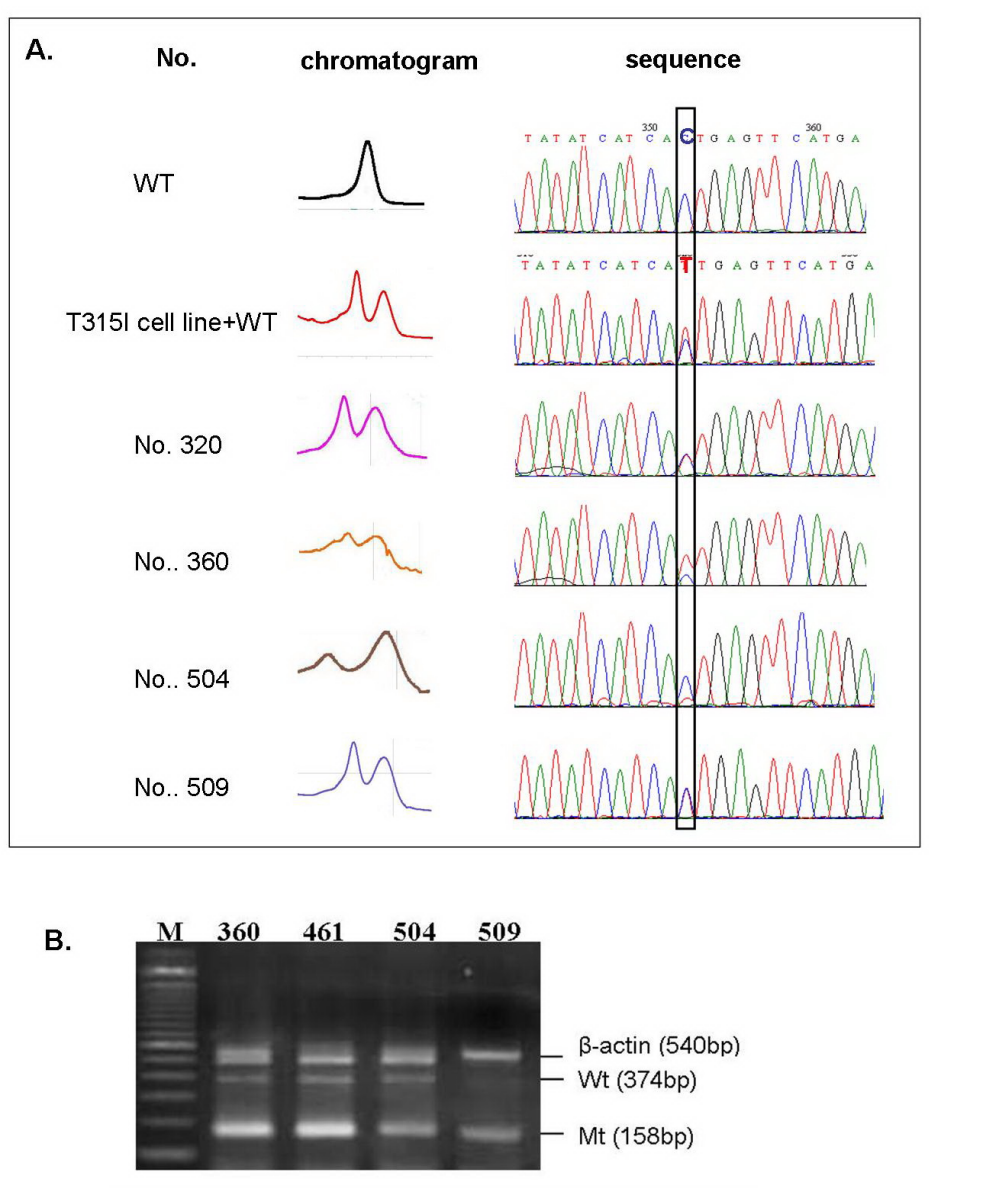
**Detection of T315I in CML patient samples by DHPLC, sequencing and AS-PCR; **Figure 3A **shows DHPLC patterns followed by sequencing analysis if a suspicious peak was observed; T315I cell lines and wild types are also shown; **Figure 3B **demonstrates representative AS-PCR results of four CML cases with abnormal DHPLC and sequencing results (patients no.360, no.461, no.504, and no. 509) **.

Nine CML patients were tested for T315 mutation using DHPLC followed by sequencing and AS-PCR. Abnormal DHPLC patterns strongly supportive of T315I mutation were observed and all were confirmed by sequencing as shown in Figure 3A. Mutant bands were comparably seen by AS-PCR. Representative AS-PCR results of four CML cases with abnormal DHPLC and sequencing results (Patients no.360, no.461, no.504, and no. 509) are shown in Figure 3B.

## 4. Discussion

Several methods have been utilized to detect the existence of *BCR-ABL KD *mutations such as direct sequencing, DHPLC, restriction fragment length polymorphism (RFLP), pyrosequencing, double-gradient denaturing electrophoresis, AS-PCR, AS real-time PCR, array based assays, and high-resolution melt curve analysis (HRM), with varying sensitivity and specificity [25,26,29-35]. In this study, we established an AS-PCR-based method to detect T315I which is the most resistant genotype associated with the highest impact on clinical outcome of CML patients. The sensitivity of our AS-PCR method was better than the sensitivities reported from most previously reported detection techniques and was slightly better than DHPLC and direct sequencing analysis in our hands. By AS-PCR, T315I mutant bands were observed in the mixtures containing as low as 0.5% of mutant alleles whereas DHPLC was unable to detect the mutants below 1.56% dilution and sequencing was unable to detect below 6.25%.

The detection sensitivity of DHPLC in our study was in the range of previously published articles (1-5%) [30,31]. Although DHPLC is considered a useful tool to screen for the presence of either known or unknown mutations, chromatograms generated from DHPLC were sometimes difficult to interpret and sequencing analysis is always needed to confirm their results. In our study, sequencing analysis was not able to detect mutant alleles below the 6.25% dilution. Therefore, it is the least sensitive method in our hands. Direct sequencing is recognized as a confirmation method for any screening tests because of its high specificity [25]. However, its disadvantage is its high costs and low sensitivity (15-25%) [26], rendering it unsuitable for routine clinical use. In terms of specificity, all methods showed no false positive results even in our triplicate experiments (0% mutant in the samples or 100% WT). Our AS-PCR method had a high specificity due to the utilization of internal mismatch primers which were designed to specifically target the mutated sequences, therefore, none of the 30 non-leukemic patient samples were falsely found to be T315I positive.

The advantage of AS-PCR is that it does not require additional post-PCR product preparations for the next step as contrasted to the multiple steps such as the DNA purification step in sequencing method and the preparation of a 1:1 mixture of mutant and WT PCR products followed by generation of heteroduplexes and homoduplexes in the DHPLC method which is much more time-consuming. AS-PCR technique can be applied in any general laboratory worldwide. Moreover, this present AS-PCR method could be performed in a single tube containing all three primers for a control gene, a WT gene, and a mutant gene; therefore, the cDNA quality could be simultaneously assessed at the same time of the detection of the WT and the mutant genes. AS-PCR is also suitable to perform in cases with a low DHPLC peak and its shape looks like a known mutation such as T315I. The disadvantage of AS-PCR is its ability to detect only known mutations using specific primer sets and optimized PCR condition for each type of mutant allele. The sensitivity of our AS-PCR method was lower than that of Roche-Lestienne C et al [20] and Kang HY et al [26], both of which did not use internal β-actin control primers. Nevertheless, we believe that the cDNA quality should be simultaneously assessed at the same time of the detection of the WT and the mutant genes, especially in the homozygous T315I mutant cases, therefore all three primers were utilized in a single-tube reaction. Current clinical practice accepts a detection method with a sensitivity of at least 0.5-1% since a higher sensitivity may detect a clone that may not be of clinical relevance [25,36]. Changes of TKI therapy based on a very sensitive molecular test may have an adverse impact if the clinical outcome is not truly affected by the presence of a minute amount of leukemic cells with that particular genetic defect.

## 5. Conclusion

Our single tube AS-PCR method is a simple, rapid, and easy to perform test which requires only simple PCR reagents and a PCR machine leading to overall lower costs as compared to other more complicated and more expensive screening methods. Detection of the most resistant leukemic clone in CML patients undergoing TKI therapy, especially those who reside in the developing worlds, should be feasible with this simple and inexpensive method. Future studies should focus on the design of other primer sets to cover other mutations associated with 2^nd ^generation TKI resistance such as V299L/F317L in dasatinib and E255K/E255V/Y253H in nilotinib.

## Competing interests

The authors declare that they have no competing interests.

## Authors' contributions

WW performed the experiments and data analysis and contributed to the drafting of the manuscript. WT performed and supervised the molecular analysis and contributed to the revision of the manuscript. CUA was responsible for the initiation and execution of the entire project and the critical revision of the manuscript. All authors read and approved the final manuscript.
